# Intérêt du clou rétrograde dans les fractures du fémur distal: à propos de 07 cas

**DOI:** 10.11604/pamj.2018.31.73.14238

**Published:** 2018-10-02

**Authors:** Redouane Hani, Mohamed Ben Aissi, Moncef Boufettal, Mohamed Kharmaz, Mohamed Saleh Berrada

**Affiliations:** 1 Service de Chirurgie Orthopédique, CHU Rabat, Hopital Ibn Sina, Université Mohammed V, Souissi, Maroc

**Keywords:** Fracture, fémur distal, ostéosynthèse, clou rétrograde, Fracture, distal femur, osteosynthesis, retrograde nailing

## Abstract

Ce travail rapporte une série de sept cas de fractures de l’extrémité inférieure du fémur traitées par enclouage centromédullaire rétrograde au service de traumatologie orthopédie au centre hospitalier Ibn Sina durant la période du 30/05/2010 au 30/04/2015. L’âge moyen de nos patients est de 54 ans avec une légère prédominance Masculine (57,1%). Les accidents de la voie publique ont représenté la principale étiologie (71,4%) et le côté droit était le plus touché (57,1%). Sur le plan opératoire, le délai moyen de l'intervention était de 2 jours. La rachi anesthésie a été réalisé dans cinq cas. La durée moyenne d’hospitalisation était de 5 jours. L’ensemble de nos patients ont consolidé dans un délai moyen de 4 mois, avec un cas de retard de consolidation à 6 mois. Nous avons obtenu de bons résultats fonctionnels avec une flexion moyenne du genou à 120°, avec un seul cas de flexion limité à 90°. Dans notre série, on avait constaté un seul cas de retard de consolidation et un seul cas de pseudarthrose. Nous n’avons noté aucun décès ni sepsis superficiel ou profond, ni complication thromboembolique ou embolie graisseuse, nos résultats étaient en général satisfaisants, ce qui nous donne le droit de dire que l’enclouage rétrograde du fémur reste une technique d’ostéosynthèse qui a toute sa place dans le traitement chirurgical des fractures distales du fémur.

## Abstract (en)

We report a series of seven cases of fractures of the distal end of the femur treated by retrograde centro-medullary nailing in the Orthopedic-Trauma Department at the Hospital Ibn Sina over the period 30/05/2010 - 30/04/2015. The average age of patients was 54 years, with a slight male predominance (57,1%). Road traffic accidents were the main cause of these fractures (71.4%) and they mostly affected the right side of body (57,1%). From a surgical point of view, the mean time between symptom onset and treatment initiation was 2 days. Spinal anesthesia was performed in five cases. The average length of stay in hospital was 5 days. The mean time of consolidation was 4 months in all patients, there was only one case of delayed consolidation (6 months). Patients had good functional outcomes with mean knee flexion 120°, with only one case of limited flexion 90°. There was one case of consolidation delay and one case of pseudarthrosis. No deaths, or superficial or deep sepsis, or thromboembolic complications or fat embolism were registered. In general we had satisfactory outcomes. Our study highlights that retrograde femoral nailing is an essential osteosynthesis technique for the surgical treatment of the distal femur fractures.

## Introduction

Les fractures de l’extrémité inférieure du fémur sont des fractures fréquentes et qui peuvent être intra-ou extra-articulaires. Elles sont considérées, comme particulièrement difficiles à comprendre et à traiter. Souvent comminutives et parfois ouvertes, touchant une articulation portante à la mécanique complexe, elles ont pour cortège un taux élevé de complications et de séquelles. L’enclouage rétrograde du fémur est une technique d’ostéosynthèse interne basée sur l’utilisation de clou centromédullaire dans le but d’obtenir une fixation solide du foyer de fracture. Les complications liées à ce traitement ne sont pas exceptionnelles, et dépendent d’une part de la complexité de fracture mais d’autre part de la réalisation de la technique elle même.

## Méthodes

Nous rapportons une étude rétrospective de sept cas de fractures de l’extrémité inférieure du fémur traitées par enclouage centromédullaire rétrograde au service de Traumatologie Orthopédie du CHU Ibn Sina Rabat durant la période du 30/05/2010 au 30/04/2015. Les patients étaient inclus dans l’étude selon les critères suivants: la survenue d’une fracture de l’extrémité distale du fémur, non pathologique, chez des patients âgés de plus de 16 ans. L’objectif de l’étude est d’évaluer et analyser les résultats fonctionnels ainsi que les complications après un traitement chirurgical par un clou rétrograde du fémur.

## Résultats

L’âge moyen de nos patients était de 54 ans avec des extrêmes allant de 30 ans à 80 ans. 71,4% de nos patients avaient moins de 65 ans. Dans notre série, on a observé une légère prédominance masculine avec 4 hommes et 3 femmes, on a noté une atteinte des hommes à un âge jeune et une atteinte des femmes à un âge avancé. Les accidents de la voie publique ont présenté la principale étiologie et ont été relevées dans 5 cas, soit 71, 4%, suivis des chutes au 2ème rang dans deux cas. Cinq patients étaient victimes d’un traumatisme de haute énergie, soit 71,4%, et 2 patients étaient victimes d’un traumatisme de basse énergie, soit 28,6%. Il y avait une ouverture cutanée punctiforme objectivée dans un seul cas, aucune lésion vasculo-nerveuse n’a été objectivée. La durée moyenne de l’intervention était de 90min, la rachianesthésie a été réalisée dans six cas, tandis que l'anesthésie générale a été réalisée dans un cas. Tous nos patients ont été installés en décubitus dorsal. La voie para-patellaire interne a été réalisée chez 6 patients, soit 85,7%, alors que la voie transe-tendineuse a été réalisée chez un patient. Dans notre série, on a utilisé un clou de type Zimmer, le diamètre des clous a varié entre 10 et 11 mm. On a utilisé des clous longs chez 4 patients, et des clous courts chez 3 patients. Tous les patients ont bénéficié d’une rééducation fonctionnelle. La durée moyenne d’hospitalisation était de 7 jours. Aucun décès n’a été déploré dans notre série et aucune infection n’a été révélée. Aucune embolie graisseuse n’a été rencontrée. Un seul cas de retard de consolidation à 6 mois a été noté. Nous n'avons signalé aucun cas de rupture de clou dans notre série. La flexion du genou était supérieure à 120° chez 6 patients, un patient avait une flexion limitée à 90°. Nous n’avons noté aucun cas de flessum ni de recurvatum ni troubles rotatoires. La reprise de l’appui a varié selon le type de fracture et l’aspect radiologique, il a été totalisé en moyenne à 3 mois. Vue les résultats fonctionnels de nos malades on peut dire que les résultats étaient satisfaisants.

## Discussion

**Figure 1:**
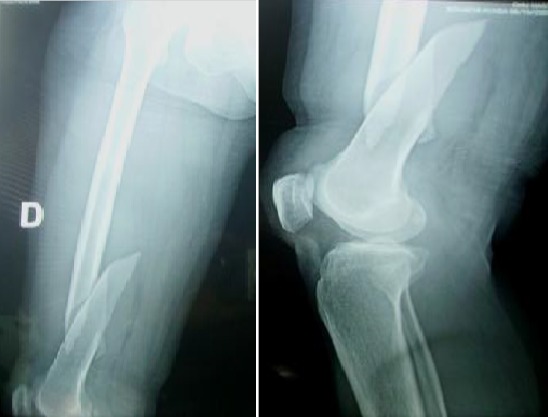
Radiographie préopératoire montrant une fracture de l’extrémité inferieure du fémur droit déplacée

**Figure 2:**
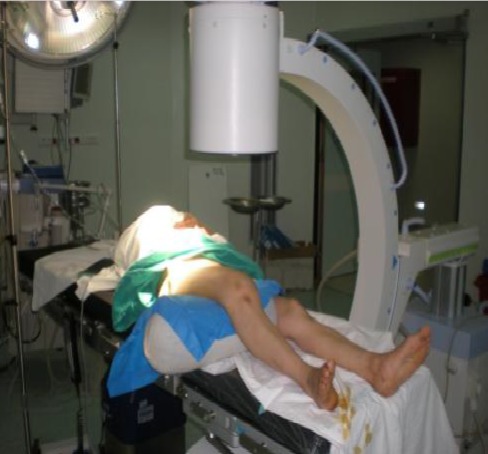
Installation du patient

Les indications de l’enclouage rétrograde sont classiques: fracture extra-articulaire, fractures articulaires simples peu ou non déplacées (Figure 1). Le patient peut être installé sur une table standard ou sur une table orthopédique. Sur une table standard le genou est fléchi à 30° grâce à un appui positionné sous le fémur distal (Figure 2). Sur une table orthopédique, la réduction est obtenue par la traction osseuse mise en place au niveau du tibia proximal, jambe légèrement pendante. Le clou doit être enfoncé suffisamment afin d’éviter tout conflit avec la patella et ne doit pas être utilisé comme levier au risque de créer un trait de fracture intercondylien. La tenue de la fixation épiphysaire peut être améliorée par l’emploi de vis avec contre-vis. L’âge moyen de notre série était inférieur à la plupart des séries étrangères. On peut expliquer ceci par l’espérance de vie prolongée chez la population occidentale et aussi par le taux élevé des accidents de la voie publique qui concerne essentiellement les sujets les plus jeunes dans notre contexte [1-3].

**Figure 3:**
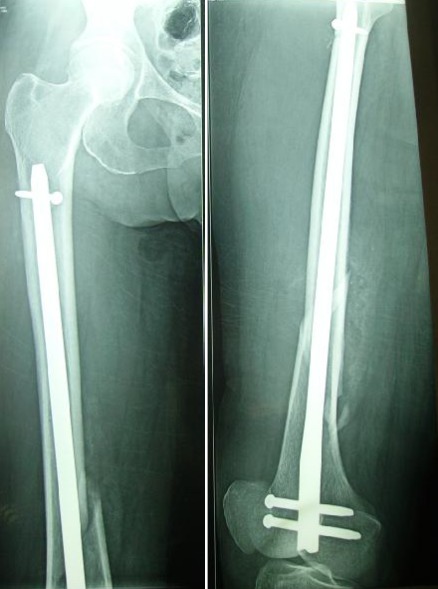
Radiographie postopératoire montrant l’ostéosynthèse par un clou rétrograde avec double verrouillage distal et proximal

Dans les séries étrangères ainsi que dans notre série, on retrouve deux populations différentes: une population de sujets jeunes, à prédominance masculine, victimes de traumatismes violents le plus souvent lors des accidents de la voie publique, et une autre de sujets âgés, à prédominance féminine, qui présentent des fractures suite à des traumatismes banals en rapport à l’ostéoporose [4, 5]. Les accidents de la voie publique (AVP) ont été la principale étiologie dans notre série. Vichard et Gurkan rapportent aussi une nette prédominance des accidents de la voie publique [1, 6]. Dans toutes les séries analysées, on retrouve une fréquence plus importante des fractures supra-condylienne par rapport aux autres types de fractures. La consolidation dans notre série rejoint les données de la littérature, il varie entre 4 et 5 mois (Figure 3).

Vue le nombre restreint des cas dans toutes les séries on ne peut pas déduire des conclusions mais d’après ces résultats on peut dire que le taux d’infection de notre série ainsi que la plupart des séries étrangères est faible ce qui est en faveur de cette technique on peut explique cela par la technique opératoire qui limite et réduit le temps de contact avec les germes [6, 7]. Les pseudarthroses du tiers distal du fémur devraient être relativement rares car il s'agit d'une zone riche en os trabéculaire qui a de bonnes propriétés ostéogéniques et dont la suppléance vasculaire est excellente. Dans la série de Vichard, on a trouvé 2 cas de pseudarthrose, soit 6,4%, le 1^er^ cas est traité par dynamisation, l’autre cas est traité par greffe osseuse [1]. Dans les séries de Ghandour et Saw, aucune pseudarthrose n’a étér évélée [5, 7]. Bocquet a retrouvé quelques cas de cal vicieux dont: 4 cas en recurvatum. 7 cas (30%) en varus ou valgus supérieur à 5° [4]. Gurkan a noté 8 cas de cals vicieux dont: 4 cas en varus. 4cas en recurvatum [6]. Sameh El-Kawy rapporte dans une série de 23 patients 9 cas de cals vicieux, dont 3 cas en varus et 6 cas en valgus [8].

**Figure 4:**
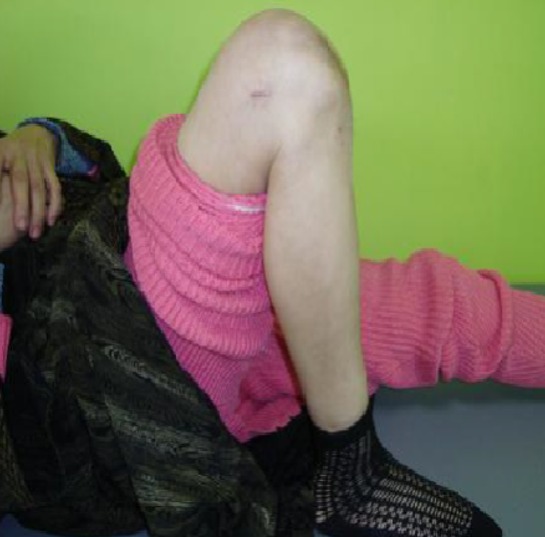
Contrôle clinique après consolidation montrant une flexion du genou à 120°

Vichard rapporte un cas de rupture de clou, soit 3,2%. Lauri Handolin rapporte 2 cas de rupture de vis de verrouillage, soit 4,5% [1, 2]. Piétu rapporte deux fractures au-dessus du matériel survenues chez des patients ostéoporotiques. Une a été favorisée par des difficultés de verrouillage proximal. Malgré la reprise par un clou plus long, l'évolution s'est faite vers une nouvelle fracture aboutissant au décès. L'autre s'est produite chez une paraplégique. [9] Le délai de consolidation dans notre série rejoint les données de la littérature, il varie entre 4 et 5 mois. Lorsque nous analysons l’ensemble de nos patients, nous constatons que 6 patients ont eu une bonne flexion du genou à 120°, un seul cas a une flexion limité à 90° (Figure 4). Gurkan rapporte une flexion à 135° chez trois patient soit 17,7%, 100 à 110° chez neuf patients soit 52,9%, 80° chez quatre patients soit 23,5%, et en dessous de 80° chez un patients soit 5,9% [6]. Dans la série de Vichard, la flexion moyenne était de 107°. Saw a constaté une flexion moyenne à 123,8°. Les séries de la littérature ainsi que notre série confirment les bons résultats fonctionnels de l’enclouage rétrograde du fémur dans les fractures de l’extrémité inférieure du fémur [1, 6, 7].

## Conclusion

Les fractures de l’extrémité inférieure du fémur sont des fractures fréquentes et relativement difficiles à prendre en charge. Nos résultats cliniques et radiologiques de notre étude étaient très encourageants, la consolidation a été survenue dans la plupart des cas dans un délai raisonnable, et le taux de complication était très faible. L’enclouage rétrograde du fémur est une alternative fiable qui mérite amplement de figurer dans l’arsenal thérapeutique des fractures de l’extrémité inférieure du fémur.

### Etat des connaissances actuelles sur le sujet

- Fractures fréquentes du sujet jeune et âgé;
- Prise en charge souvent difficile;
- Complications assez fréquentes.

### Contribution de notre étude à la connaissance

- Le clou rétrograde est l’option de choix de l'ostéosynthèse des fractures sus et intercondyliennes du fémur distal;
- Il assure une fixation solide et stable;
- Avec de meilleurs résultats fonctionnels.

## Conflits d’intérêts

Les auteurs ne déclarent aucun conflit d'intérêts.
